# Assessing the suitability of the Carer Support Needs Assessment Tool (CSNAT-Paediatric) for use with parents of children with a life-limiting condition: A qualitative secondary analysis

**DOI:** 10.1177/02692163231214471

**Published:** 2023-12-23

**Authors:** Victoria Fisher, Karl Atkin, Gail Ewing, Gunn Grande, Lorna K Fraser

**Affiliations:** 1 Department of Health Sciences, University of York, York, UK; 2 Department of Sociology, University of York, York, UK; 3 Centre for Family Research, University of Cambridge, Cambridge, UK; 4 School of Health Sciences, Faculty of Biology, Medicine and Health, The University of Manchester, Manchester, UK; 5 Florence Nightingale Faculty of Nursing Midwifery and Palliative Care, Cicely Saunders Institute, King’s College London, London, UK

**Keywords:** Paediatrics, palliative care, qualitative, child

## Abstract

**Background:**

The demands of caring for a child with a life-limiting condition can have a profound impact on parents’ health and wellbeing. Currently, there is no standard procedure for identifying and addressing the support needs of these parents.

**Aim:**

To assess the suitability of the Carer Support Needs Assessment Tool (CSNAT (Paediatric)) for use with parents of children with a life-limiting condition.

**Design:**

Secondary qualitative content analysis of two qualitative datasets exploring the health, wellbeing and experiences of support of mothers and fathers of children with a life-limiting condition.

**Setting:**

A total of 30 mothers and 12 fathers were recruited via four UK children’s hospices and social media.

**Results:**

Parental experiences of support mapped onto the existing domains of the CSNAT (Paediatric). One aspect of their experience, surrounding their child’s educational needs, went beyond the existing domains of the CSNAT. An adapted version of the tool CSNAT (Paediatric) should include this domain.

**Conclusion:**

The CSNAT (Paediatric) is a relevant tool for the assessment of parental support needs. Further research should assess the acceptability and feasibility of implementation of the broader intervention: CSNAT-I (Paediatric).

**What is already known about the topic?**

- The parents of children with a life-limiting condition provide extensive care for their child at home and must be well supported to do so.
- The support currently available for these parents is limited, with no standard means of identifying and addressing their needs.
- A process through which these needs can be both identified and addressed is needed.

**What this paper adds?**

- The existing domains of the CSNAT (Paediatric), and an additional domain surrounding education, are applicable to the potential support needs of parents of children with a life-limiting condition.

**Implications for practice, theory or policy**

- Results demonstrate the applicability of the CSNAT to the needs of parent caregivers.
- The next stage of this research should assess the feasibility of the intervention in which the tool sits.

## Introduction

The number of children living with a health condition that may shorten their life (hereafter life-limiting condition) is increasing.^1^ This increase has been reflected across national and international contexts,^2,3^ with international research agendas centralising the importance of understanding the needs of caregivers.^4^ The importance of such is also emphasised by the World Health Organization.^5^ It is the parents of these children that commonly provide all their care, which can be extensive. Medication management, gastrostomy management, ventilation, advocacy in healthcare and education, transport and extensive appointment schedules are just some of the responsibilities that must be met by these parents. This is alongside other general parenting responsibilities and looking after the needs of other family members.^6^

The social, emotional, psychological and financial impacts of this on parents are described in existing literature.^7,8^ Mothers have a higher incidence of common mental and physical health problems, as well as premature morbidity, compared to other mothers.^9^ The need for further research on appropriate support and interventions for both mothers and fathers has been recognised as a priority.^10,11^ However, despite this recognised priority and subsequent research efforts, there is no process in place that is able to systematically identify and address parents’ support needs in practice. Support depends largely on individual relationships with professionals.^12^ Research highlights the need for a process that is able to identify the needs of parents and for interventions to improve their wellbeing.^6^

An intervention that has been shown to be effective for caregivers of adults with palliative care needs is the Carer Support Needs Assessment Tool intervention (CSNAT-I).^13–15^ This intervention is a person-centred process to support carers, with the collaboration between carer and practitioner at the forefront if its function. A question-and-answer evidence-based assessment tool (CSNAT) sits within a five-stage process (CSNAT-I); (1) introduction to the tool; (2) carers’ consideration of needs; (3) assessment conversation; (4) shared action plan and (5) shared review. The tool itself contains two groupings of domains related to the support needs of carers. Domains are either ‘direct’ (i.e. related to support that parents need for their own health and wellbeing), or ‘enabling’ (i.e. related to support needed to enable them to care for their child).

The tool has been adapted for carers of adults with long-term conditions (Motor neurone disease, Chronic obstructive pulmonary disease)^16–18^ and subsequently for use with family caregivers of children with a rare condition.^19,20^ Existing studies have aimed to assess the relevance and applicability of the tool in a paediatric population.^19^ However, its initial adaptation was carried out with a very small sample of parents and stakeholders in the context of the United States healthcare system.^20^ Therefore, this study aimed to assess whether the domains of the CSNAT (Paediatric) tool, summarised in Table 1, are suitable for use with parent caregivers of children with a life-limiting condition within the UK, using existing qualitative datasets.

**Table 1.** Summary of domains included in the CSNAT (Paediatric).

Grouping	Domains included in each group
‘Direct’ domains (support needed by caregivers to look after their own wellbeing).	- Having time for yourself during the day - Getting a break from caregiving overnight - Looking after your own health (physical problems) - Financial, legal or work issues - Practical help in the home - Dealing with your feelings or worries - Your beliefs and spiritual concerns - Taking care of others in the home (e.g. siblings, older parents, grandparents) - Relationships with spouse/ partner
‘Enabling’ domains (support needed by caregivers to enable then to care for their child).	- Understanding your child’s illness - Managing your child’s symptoms including giving medicines - Providing personal care for your child, for example dressing/washing - Knowing who to contact if you are concerned about your child (for range of needs including at night) - Equipment to help care for your child - Talking with your child about his/her illness - What to expect in the future when caring for your child

## Methods

### Study design

This study utilised two primary qualitative datasets exploring the health, wellbeing and experiences of support of mothers (dataset 1) and fathers (dataset 2) of children with a life-limiting condition. These studies are described below along with summaries in Table 2.

**Table 2.** Summary of primary datasets.

Dataset	Description	Aim of original study	Recruitment	Data collection	Data analysis	Number of participants
Dataset 1^12^	Mothers of children with a life-limiting condition	To explore the health and wellbeing of mothers of children with a life-limiting condition, their experiences of accessing healthcare for themselves, factors that affect their health and ways in which they look after their health.	3 UK children’s hospices, social media	Semi-structured interviews carried out between November 2020 and June 2021	Thematic analysis	30
Dataset 2	Fathers of children with a life-limiting condition	To explore the caregiving experiences and health and wellbeing of fathers of children with a life-limiting condition.	3 UK children’s hospices, 1 NHS site and social media	Semi-structured interviews carried out between November 2021 and March 2023	Thematic analysis	12

### Participants

For the respective primary studies, mothers (dataset 1) and fathers (dataset 2) were eligible if they were 18 years or above and their child was aged 25 years or under and had been diagnosed with a life-limiting condition.

### Sampling

Purposive sampling for each study was undertaken by healthcare professionals during their regular means of contact with parents as per the inclusion criteria above.

#### Recruitment

For each of the primary studies, participants were recruited through four UK-based children’s hospices, an NHS children’s hospital and via social media (as per Table 2). If eligible, healthcare professionals invited mothers/fathers to the studies and provided them with an information sheet. If they wanted to take part in an interview, participants gave their consent for a member of the study team to contact them directly. The study team then got in touch with potential participants, confirmed eligibility, answered any questions and booked an interview if appropriate. Potential participants who had seen the study details on social media were able to contact the study team directly and the same steps were followed.

### Data collection

30 interviews were conducted with mothers (dataset 1) between November 2020 and July 2021, and 12 with fathers (dataset 2) between November 2021 and March 2023. Each of these original datasets were collected via voice recorded semi-structured interviews via Zoom or over the telephone, using open ended questions, to explore the health and experiences of mothers and fathers. This included accounts of their own mental and physical health, their experiences of looking after their health including how and when they access health services/support, things that affect their health adversely and ways in which they felt they could look after their health, all in the context of being a caregiver to a child with a life-limiting condition. Drawing on an interpretivist/constructivist paradigm, we explored parents’ lived experiences alongside the recognition that our own assumptions and interpretations shape the research process and findings.

### Data analysis

For the primary studies, data was analysed using reflexive thematic analysis.^21^

### Secondary analysis

Secondary analysis of these datasets was carried out using qualitative content analysis.^22^ The consolidated criteria for reporting qualitative research (COREQ) guidelines for reporting qualitative analyses were followed as far as they apply to secondary analysis.^23^ The secondary analysis of the data was undertaken to further assess whether these parents’ support experiences and needs were accounted for in the existing domains of the CSNAT (Paediatric) or whether domains needed to be amended or new domains added. Both datasets (anonymised transcripts of interviews with mothers and fathers and coding structures from primary analyses) were combined, and qualitative content analysis was conducted.^22^ The initial inductive coding structures from the primary analyses were used in this secondary analysis; a directed content analysis^22^ to consider the experiences and needs of parents in relation to the CSNAT (Paediatric). This is appropriate given that the secondary analysis relates to, as the primary analyses did, the health experiences and support needs of parents and that the same authors conducted the data collection and analysis.^12^

Data were mapped onto the existing domains of the CSNAT (Paediatric) according to latent and semantic meaning. Data that did not relate directly to the domains within the CSNAT (Paediatric) were categorised separately. These codes were discussed with the wider research team to decide whether they could be categorised through said discussion, or whether a new domain needed to be identified. The data that each code pertained to was checked once categorised. All coding and categorisation was discussed with the wider team members.

## Ethical considerations

Research Ethics Committee approval was granted for the primary studies (reference 20/NE/0164 and reference 21/LO/0591) on 2nd July 2020 and 17th November 2021. These approvals allow for secondary analysis.

## Patient and public involvement

A family advisory board made up of a group of parents and carers with experience of paediatric palliative care, assisted with all stages of the primary study. For this study, the revised CSNAT (Paediatric) was discussed with the Family Advisory Board. Discussions surrounded the appropriateness of the domains and of examples of support needs included within the tool. The group also discussed implementation of the broader intervention (CSNAT-I), which will be considered in the next phase of this programme of research.

## Results

interviews with 30 mothers (of 34 children) and 12 fathers (of 15 children) were included in this secondary analysis. Mothers were aged between 32 and 60 years and fathers were aged between 39 and 51 years. Each group resided across various regions of the UK. This included parents who did and did not have support from a children’s hospice. Child characteristics are provided in Table 3.

**Table 3.** Child characteristics.

Dataset	Sex of child *n* (%)	Age range of children (mean age)	Diagnosis
Dataset 1	Male 16 (47%) Female 18 (53%)	1–23 years (10.8 years)	Diagnoses included: congenital (2.9%), genetic (55.9%), neurological (26.5%) and cardiac (2.9%) conditions
Dataset 2	Male 7 (48%) Female 8 (52%)	3–23 years (11.4 years)	Diagnoses included: congenital (13.3%), genetic (33.3%), neurological (40%), metabolic 6.7%) and cardiac (6.7%)

Findings related to parents’ support needs are discussed below, along with exemplar quotes from participants. Further examples of aspects of parents’ support needs are presented next to the individual domains of the tool in Tables 4 and 5. The results are broken down into (1) domains surrounding the direct support needs of caregivers (Table 4) and (2) domains surrounding support to enable parents to fulfil their caregiving role, as per the structure of the tool (Table 5). This is followed by findings surrounding an additional domain identified; the educational needs of the child.

**Table 4.** Support needs as they relate to direct domains of the CSNAT (Paediatric)^20^ tool.

Support needs domain (direct support needed by caregivers to look after their own wellbeing)	Key aspects related to met/unmet support needs or helpful input provided
Having time for yourself in the day	• Caring for a child with a life-limiting condition is like a full-time job. • There is no break from caregiving. • Parents spend a lot of time chasing up professionals/services over the phone. • Even when parents do have time for themselves, there is constant worry about their child. • Full-time work followed by caregiving responsibilities at home in the evening/overnight.
Getting a break from caring overnight	• Parents suffer from a lack of sleep. • Child requires constant monitoring, including overnight. • Respite support would be helpful, though it needs to be with highly trained professionals to enable trust in parents. • Parents must be constantly alert to any change in child’s condition, making it difficult for them to fall asleep. • Even when there is care in place overnight, parents struggle to sleep due to stress.
Looking after your own health (physical problems)	• Physical impact of caregiving including back problems from lifting and exhaustion from a lack of sleep. • A lack of time to think about own health, including lack of time for exercise. • Difficult to find time to prepare healthy meals. • Difficult to access GP appointment, particularly as there is limited time to address own needs. • Physical health concerns often immediately attributed to caring responsibilities by professionals. • Worried about becoming unwell in the future. • Find it difficult to discuss own needs with those closest.
Your financial, legal or work issues	• Unable to work; caregiving is fulltime. • Workplace is understanding and flexible; flexibility is imperative. • Workplace do their best to be supportive but no-one truly understands what caregiving entails. • Needed a career change due to child’s illness. • Adapting to a different financial situation was challenging. • Paid for own house adaptations/ equipment.
Practical help in the home	• Parents discussed a need for more practical help in the home. This would enable them to find time for regular activities such as having dinner together or spending more time with other children. • Practical help completely disappeared during the COVID-19 pandemic. • There is a lack of time to complete regular household tasks. • Practical help would enable family time.
Dealing with your feelings and worries	• Finding a shared understanding with others is useful for practical and emotional guidance. • Psychological impact of caregiving; anxiety, stress, depression and tiredness. • It is difficult to access timely and appropriate psychological support. • There is a lack of understanding from healthcare professionals surrounding the type of psychological support needed. • Having the ability to pay for own psychological support is helpful. • Parents do not want to be judged as not coping; they can find it difficult to ask for help and often wait until a crisis. • Being honest about feelings is particularly easy at children’s hospice. • There is a need to be strong for others, including child. • Sometimes, there is no time to address their own feelings and worries due to the demanding and urgent nature of caregiving. • Exploring feelings is often unhelpful for parents who must focus on caregiving.
Your beliefs and spiritual concerns	• Finding comfort in religious beliefs.
Relationship with spouse/ partner	• Don’t want to burden partner with concerns, particularly if partner is struggling. • Parents discussed the need to work together as part of an established routine.

**Table 5.** Support needs as they relate to enabling domains of the CSNAT (Paediatric)^20^ tool.

Support needs domain (enabling domains to help them support their child)	Key aspects related to met/unmet support needs or helpful input provided
Understanding your child’s illness	• There is a distinct lack of support for rare conditions, particularly at diagnosis. • Parents often have to conduct own research due to shortcomings in information/ communication from professionals. • Diagnosis took a long time with little information in the interim.
Managing your child’s symptoms including giving medicines	• Time consuming care takes up most of the day. • Family members are unable to help with complex medical procedures. • Multiple medications must be managed, which is extremely stressful. • Managing pain is particularly challenging. • If parents demonstrate competence, they are given even more responsibility. • Parents must be constantly alert to a change in child’s condition/symptoms, including overnight. • Obtaining repeat prescriptions can be challenging. • Do not trust anyone else to provide care for child.
Providing personal care for your child, for example dressing/washing	• Most procedures, including washing, are extremely time consuming. • Not possible to bathe child alone. • Washing is particularly difficult given a lack of equipment.
Knowing who to contact if you are concerned about your child (for range of needs including at night)	• Parents are tired of re-explaining their child’s condition to professionals. • There is a lack of support/knowledge from general practitioners. • Parents described having to attend hospital emergency department when their child was unwell due to a lack of support elsewhere. • Children’s hospices are able to act as point of contact for concerns.
Equipment to help care for your child	• Parents often have to fight for appropriate equipment and endure long waits in obtaining it. • Families live in unsuitable housing without appropriate adaptations. • Parents have health problems as a result of lack of suitable equipment that is hoists.
Talking with your child about his/her illness	• Parents have to make decisions surrounding how much they tell their child about their condition and prognosis.
Taking care of others in the home (e.g. siblings, older parents and grandparents)	• Parents do not want to burden others, including partner and children. • Parents also had concerns about how much to tell siblings. • Parents worry about siblings, including their informational needs; siblings often have very different needs to unwell child. • Worried about other family members needing care, for example grandparents.

### Direct domains

Parents discussed support needs in relation to their own health and wellbeing. They discussed examples of when they felt they had felt able to look after their own health and wellbeing, as well as instances in which they felt that they lacked the ability or support to do so. Parents felt that professionals could often misunderstand their experiences and support needs. This made it difficult to access appropriate and timely support. In particular, parents felt that their experiences of stress could be mis-labelled as mental illness. This was particularly evident when parents were prescribed short courses of psychological therapies for anxiety or depression, or prescribed antidepressants, both of which were unable address the unique set of challenges faced by parent caregivers, which were more socially located:

*‘There was about four of five months where there were quite difficult conversations with the social worker. . . it was felt that our distress was a danger to [our child] because it meant that we were less likely to be coping as a family and therefore needed to be fixed with antidepressants, as if that was going to fix it’ [father 11].*

Parents further highlighted the need for a distinction between the normal distress related to their child’s condition and clinical depression in need of a specific treatment.

*‘I really try to distinguish between distress and depression where the circumstances I’m in are naturally going to be producing distress and to not feel like that’s the wrong thing but to also try to watch out for feeling more low than the situation warrants’ [father 10].*

Getting a break from caregiving was important for the health and wellbeing of parents. Parents were physically and emotionally exhausted because of the lack of opportunity to rest, particularly evident in parents that had to monitor their child overnight. This also impacted upon their relationship with their partner/spouse, often each taking alternate ‘night shifts’. Overnight breaks were highly valued by parents, as long as their child was with a trusted and trained professional or family member. Hospice respite support was particularly valuable in allowing parents to feel safe in the knowledge that their child was being well cared for.

‘*I think he went for about four days on his own, which gave me a break. It was better for me knowing that the nurses were there if he took a turn, or if they needed me, they could just ring me. And, to be fair, I think I’ve rung them more than I’ve rung my mum this week, just to check on him. But it’s a well supported place, so you can’t fault them’ [mother 14].*

Getting a break during the day was not always possible. Even when their child attended school, parents described spending their days on more administrative tasks associated with their child’s care, including chasing up services for appointments, medication and equipment.

‘*And we spent all or days on the phone ringing this one and that one and yes, we were parents of course we want to look after our kids, we haven’t got time for this, if you guys were doing your job properly we wouldn’t have to do this’* [father 10].

The value of workplace support was highlighted by parents. Most primary caregiving parents found it difficult to maintain employment alongside caregiving, though those with support and flexibility at work were able to attain some balance. Parents had financial concerns as a result of changes to their employment, to accommodate their caring responsibilities, which were particularly evident in fathers’ accounts.

*‘I gave up work, we agreed to do it, I get carer’s allowance, we’ve got 4 children with special needs so one parent can only claim for one child, so I claim for one of the others and it’s £67 a week and I’m doing full nursing care twice a day every day for 5 days out of 7’ [father 1]*.

Parents described physical health problems as a result of not having the appropriate medical equipment to care for their child. They often neglected their own physical health as a result of not having enough time to make/attend medical appointments or feeling that their own health was a priority until medical attention was critical.

*‘Obviously, the children are getting quite big now, they’re quite heavy to carry and I’m struggling to pick them up. I’m struggling to walk around. It was just getting to the point that it had been 6 months and I was thinking you know what I need to see somebody now. I saw a GP yesterday actually and they’ve basically said there’s not a lot they can do, just kind of do some exercises and there isn’t much they can do’ [mother 2]*.

Parents described feeling exhausted and overwhelmed by the constancy of their roles. They highlighted the absence of any practical help in the home, meaning that they spent any free time completing ‘regular’ household tasks.

‘*So once they’re sorted then obviously we do the regular parenting stuff of shopping, answering emails s*o for me, yes, there is no rest*’* [father 1].

A central thread through each of these direct domains was the constancy of the responsibilities associated with caregiving; including the lack of time to engage in anything other than caregiving, which had serious implications on parental health and wellbeing. Further aspects of direct met/unmet support needs are summarised below.

## Enabling domains

Parents also discussed the support needed to enable them to provide care for their child. They often had to adapt to their caregiving responsibilities very quickly, learning new techniques and managing numerous medications. For those whose children had rare conditions, they often gathered information through their own research via the internet. Parents highlighted the need for more support and information from healthcare professionals at the time of their child’s diagnosis. Many of these children were diagnosed during the COVID-19 pandemic which further exacerbated communication and support issues with healthcare professionals and availability of information.

*‘Just as we got the diagnosis, the pandemic hit and so [children’s hospital] gave us the diagnosis just as they were heading out the door because they were evacuating the building so we were left to Google and find out about the condition that way. There wasn’t much available’ [father 11].*

Accessing suitable equipment could be a real challenge for parents, often facing long waits and battles for such. Some parents had set up fundraising schemes or bought equipment themselves as to avoid these ‘battles’. However, for some self-funding was not an option and they described stress as a result of having to understand how to navigate and challenge the system in order to get appropriate support and equipment for their child.

*‘We have really struggled, my god. It’s taken a year to get any support from child services. It feels like every professional who we see bar some exceptions is so focused on trying to keep [child] off their caseload and trying to reduce the amount of exposure they have to her level of need, no doubt in terms of self-preservation of their service. So it’s been very hard’ [father 11].*

Parents found it difficult to know who to contact if they were concerned about their child. They often had to resort to the hospital emergency department. Parents described issues with having to re-explain their child’s condition and needs to multiple healthcare professionals. This not only added to the time required to obtain appropriate care for their child but also adding strain to their own emotional wellbeing.

*‘Because otherwise you ring up and you get the on-call doctor and they’re just . . . I mean the notes must be full of information, but they don’t ever seem to know anything about them and when your first question is, are they on any medication, you’re thinking . . . it’s just exhausting going through it again and again and again with different people and especially if you’re worried about your child and you’re ringing the doctor, you just need to be able to get some answers, rather than having to go through the whole medical history’ [mother 18].*

Knowing how to discuss things with their child or how much to tell them was difficult, particularly when parents considered the psychological effects on their child.

*‘She’s got very traumatised by hospital admissions and she’s got horrendous separation anxiety and she just follows me around the house. She sleeps in our bedroom. . . She was a normal six year old who quite happily went to bed, read a bit, pottered round her bedroom and would put herself to bed between 8:00 and 9:00 and you’d go up and 9:00 and she’d be fast asleep. There’s no way that happens now. Evenings mummy has to be in the bedroom before she’ll fall asleep. I get why that’s happened’* [mother 21].

Similarly, parents discussed concerns surrounding how much to tell siblings who were often deeply affected by their sibling’s condition and its impact upon family life.

*‘She comes into our room in the middle of the night, saying, “Is she alive? Is she dying?” yes it’s really hard for her. Really, really hard. She’s intelligent enough to understand that there’s a very big problem that can’t be fixed but she is only ten. So she doesn’t have the maturity to deal with it. As if anybody can deal with a child dying, but yes it’s hard when you’re ten’* [mother 2].

Some parents described good sibling support, particularly at children’s hospices. However, those without connections to a hospice found it difficult to find appropriate support for their other children, not only due to concerns about their general wellbeing but also the possibility that siblings were genetic carriers of the same conditions.

*‘His sister has been diagnosed as a carrier of Duchenne as well and there wasn’t any support for her either. And obviously she knows more about it than any of the others really. It would have been nice to have something for her to say, you know, it’s going to be okay’ [mother 7].*

### Additional domains

Data mapped well onto the existing domains of the CSNAT (Paediatric). One aspect of parents’ experiences, not captured by these domains, was the issue of accessing appropriate education for their child. Parents highlighted instances in which they felt their child was being well supported by the school.

*‘When he was in the infant school, I'd find myself going to pick him up and they'd be saying, we felt [child 10] was struggling with this so we've done this and it's fine now so they were kind of finding problems, solving them and then by the way, we've sorted that out’ [mother 10].*

Finding an appropriate educational setting was extremely important for parents although this could be challenging, particularly for parents of children with complex needs. Ensuring that the school were trained in procedures, such as feeding, could be difficult.

*‘She’s working towards doing full days, but school need to be trained to do her tube feeds because if she stays all day they’d have to change the milk bottle in the middle of the feed. So there’s a whole training programme that they have to do with the [hospital] community nurses and they aren’t particularly organised about coming out and organising training for the school’ [father 8].*

Having appropriately trained staff in place was essential, including on the journey to school if the child received transport.

*‘As she’s got older as well we do have a care package from both Health and Social Services. For example, to get to school she’s entitled to a school bus so she gets on the bus every morning but she needs to have a carer with her for that because of her unsafe digestion which can be an issue’* [father11].

## Discussion

This paper discusses the experiences and support needs of parents of children with a life-limiting condition in relation to the domains of the CSNAT (Paediatric) tool. The initial paediatric adaptation of this tool, as mentioned, was carried out with a small sample of stakeholders and in the context of the US healthcare system.^20^ Despite encouraging feasibility results in Australia,^19^ it was not clear whether this relevance could be entirely upheld in other contexts, that is in the UK. It was also unclear whether the included domains sufficiently covered potential parental support needs, having been developed with such a small sample of parents (*n* = 10).

This mapping study has demonstrated that the experiences and support needs of both mothers and fathers map well onto the ‘direct’ and ‘enabling’ domains of the tool and through the addition of an extra domain relating to education, the tool is further able to represent all the potential support needs of these parents. As with the original version of the CSNAT,^14,24^ the domains of the CSNAT (Paediatric) tool are meant to encompass all the areas within which parents may have need for support, informed by issues that we know are relevant to parental caregiving, and provide the opportunities for discussion of areas where more support is needed. The tool is meant to be used within a structured process, CSNAT-I (Paediatric), which allows parents to identify and prioritise domains where they need more support, and discuss with a professional their individual support needs within prioritised domains and what supportive input would help to address these needs.

Central to parents’ accounts of their own health and wellbeing, are tensions in prioritising their own needs alongside the needs of their child. The CSNAT Intervention (CSNAT-I) helps with addressing this issue, allowing parents to feel recognised as caregivers as well as individuals whose own health and wellbeing is important. Given that the CSNAT (Paediatric) tool has been shown to be appropriate and comprehensive, the next stage is to assess the acceptability and feasibility of implementing and evaluating the full CSNAT-I (Paediatric) in this population in the UK healthcare setting.

## Strengths and limitations

Existing literature surrounding the support needs of parents is mainly based upon the perspectives of mothers, and so the inclusion of in-depth interview data from fathers is an important contribution. Whilst the sample was diverse enough to represent different conditions, ages of child and time since diagnosis, the majority of the sample were of white British ethnicity. Participants for this study were recruited via UK based children’s hospices and via social media. However, much of the sample were recruited by hospice staff meaning that there may be an underrepresentation of those who do not receive support from a children’s hospice, which is quite often the case for this population.

## Implications for practice

These results demonstrate the suitability of the CSNAT (Paediatric) tool for use with parents of children with a life-limiting condition and that an adapted version of the tool for the UK should include a domain surrounding educational needs of the child. Further research is needed to assess the acceptability and feasibility of the broader CSNAT (Paediatric) Intervention in a UK setting which will form the next stage of this study. It may be that further adaptations are required in other cultures and contexts for the future.
